# A Preliminary Examination of the Impact of Working Memory Training on Syntax and Processing Speed in Children with ASD

**DOI:** 10.1007/s10803-021-05295-z

**Published:** 2021-11-01

**Authors:** Hélène Delage, Inge-Marie Eigsti, Emily Stanford, Stephanie Durrleman

**Affiliations:** 1 Department of Psycholinguistics and Speech-Language Therapy, Faculty of Psychology and Educational Sciences, University of Geneva, 28 Blvd. Pont d’Arve, 1205 Geneva, Switzerland; 2 Department of Psychological Sciences, University of Connecticut, Storrs, CT 06269 USA; 3 Department of Medicine, Faculty of Science and Medicine, University of Fribourg, 5 Chemin du Musée, 1700 Fribourg, Switzerland; 4 Present Address: Department of English Language and Literature, Faculty of Humanities, University of Geneva, 12 Blvd. des Philosophes, 1205 Geneva, Switzerland; 5 Department of Linguistics, Faculty of Humanities, University of Geneva, 2 rue de Candolle, 1211 Geneva 4, Switzerland; 6 Department of Psychology, Università degli Studi di Milano-Bicocca, 1 Piazza dell’Ateneo Nuovo, 20126 Milano, Italy

**Keywords:** Autism spectrum disorder, Children, Working memory, Training, Syntax, Attention

## Abstract

In addition to deficits in pragmatics, children with autism spectrum disorders (ASD) have weaknesses in complex syntax and working memory (WM). These two deficits may be closely related. Previous work investigated the effects of WM training in developmental language disorders and showed significant improvement in both WM and syntax. The current study tests the impact of 12 h of WM training across 8 weeks in 30 children with ASD, aged 5 to 11. Results showed direct improvements on untrained WM tasks, as well as transfer effects to syntax and processing speed. Stronger WM led to better syntactic abilities. While they must be replicated, these exciting results provide impetus for further studies of WM interventions.

**Supplementary Information:**

The online version contains supplementary material available at 10.1007/s10803-021-05295-z.

Autism spectrum disorder (ASD) is a neurodevelopmental condition characterized by persistent deficits in social communication and social interaction, as well as restricted and repetitive patterns of behavior, interests and activities (DSM-5, American Psychiatric Association, 2013). Language performance of children with ASD is highly variable, ranging from no language at all to fluent speech (Eigsti et al., 2011; Lim, 2018; Wan et al., 2011). Children with ASD present intellectual disabilities in 30–50% of cases (Centers for Disease Control and Prevention, 2016), but contrary to popular belief, their language performance is not necessarily dependent on their IQ level (Durrleman & Delage, 2016; Kjelgaard & Tager-Flusberg, 2001). Thus, children with a low IQ may have language skills in the normal range while others may have language deficits with a normal non-verbal IQ, as is typically the case of children with Developmental Language Disorder (DLD).^1^ There is a consensus that pragmatic impairments are highly prevalent in individuals with ASD (Tager-Flusberg, 1996), and that these relate to core deficits in theory of mind (Baron-Cohen et al., 1985; Khimi, 2014). However, other areas of language may also be impaired, such as phonology (Wolk et al, 2016), lexicon (Kjelgaard & Tager-Flusberg, 2001) and morphosyntax (Brynskov et al., 2017; Durrleman & Delage, 2016; Durrleman et al., 2016; Oi, 2008, 2010; Riches et al., 2010; Silleresi et al., 2018; Terzi et al., 2014; Tuller et al., 2017; Zebib et al., 2013). Some 60 to 70% of children with ASD perform similarly to children with DLD on tasks assessing lexicon (Kjelgaard & Tager-Flusberg, 2001), phonology (Zebib et al., 2013) and morphosyntax (Durrleman & Delage, 2016; Silleresi et al., 2018). These findings have been interpreted to suggest a comorbidity between ASD and DLD, sometimes attributed to a shared etiology and common risk genotype (Bishop, 2010; but see Williams et al., 2008). High co-morbidity between the two populations suggests that training that has been shown to be effective in children with DLD could be equally beneficial in ASD, prompting the current study in which we train working memory (WM) in participants with ASD and observe transfer effects to complex syntax, as already demonstrated in children with DLD (Delage et al., 2020, 2021; Stanford et al., 2019).

## Working Memory in ASD

Both ASD and DLD involve difficulties in executive functions (see McCrimmon et al., 2016 for ASD; Kapa & Plante, 2015, for DLD), in particular in WM. WM is defined as the temporary storage and manipulation of information needed to perform complex cognitive tasks related to learning, reasoning and language processing (Baddeley, 2003). Although various WM models exist, such as Cowan (1999), Miyake et al. (2000), Engle (2002) or Barrouillet and Camos (2012), Baddeley’s tripartite, multi-component, model of WM remains highly influential in psycholinguistics. This model integrates an attentional control system, the central executive and two subsystems: a phonological loop that stores and manipulates acoustic and verbal information, and a visuo-spatial sketchpad that stores and manipulates visual and spatial information.

The capacities of the phonological loop are assessed by simple-span verbal tasks (Barrouillet & Camos, 2007). These tasks require simple maintenance and recall of verbal information (e.g., forward digit span, word and nonword spans). Simple spans can be further subdivided into item and serial order short-term memory (Majerus et al., 2006, 2009). Item memory refers to the storage of lexical items, including their semantic and phonological representations; serial refers to the order in which the items are presented. The capacities of the central executive are measured using complex-span tasks that typically add a dual or interfering task to a memory task, for example by asking participants to evaluate the truth value of a series of sentences, and then recall the final word in each sentence (Barrouillet & Camos, 2007). Backward digit span also belongs to this category (Redick & Lindsey, 2013), although whether it differs from forward digit span remains controversial (St Clair-Thompson, 2010).

Both simple and complex-span tasks reveal WM deficits in ASD (Alloway et al., 2009, 2016; Bennetto et al., 1996; Eigsti, 2009; Gabig, 2008; Joseph et al., 2005; Schuh & Eigsti, 2012; Williams et al., 2006). Schuh and Eigsti (2012) found deficits in 18 English-speaking participants aged 9–17, with high-functioning ASD, in nonword repetition (simple span) as well as in listening recall task (complex span). In 21 French-speaking children and adolescents with ASD aged 5–16, Durrleman and Delage (2016) reported verbal WM impairment on both types of spans, on nonword repetition and forward as well as backward digit span. Despite occasional reports of preserved WM capacities in this population (see for example Alloway, 2018), a meta-analysis conducted by Habib et al. (2019) on 34 studies of children and adults with ASD (*n* = 226 in total) confirmed deficits in both verbal and visuospatial components of WM, where neither age nor IQ explained the observed WM differences. Another meta-analysis published by Wang et al. in (2017) reached the same conclusion, for both simple and complex spans. A meta-analysis which focused on more general executive functions in ASD (Demetriou et al., 2018) also pointed to a broad executive dysfunction in ASD, including WM deficits that were relatively stable across development.

## Attentional Capacities in ASD

Although WM and attentional systems are conceptualized as distinct cognitive structures, they are closely related. An attentional component is included in all WM models (Baddeley, 2003; Barrouillet et al., 2004; Cowan, 1999); in Baddeley’s model, the central executive is an attentional-controlling system which coordinates the more passive subsystems. Taking a developmental perspective, Garon et al. (2008)’s integrative framework model of executive functions includes a selective attention system which supports the further development of higher cognitive functions such as WM, inhibition and shifting. In this model, *selective attention* is a lower-order skill which consists of focusing attention on relevant stimuli while ignoring distracting information. Selective attention is typically assessed by asking subjects to identify visual or auditory target stimuli under various distracter conditions, as in the Test of Everyday Attention for Children (TEA-Ch, Manly et al., 1999). Higher-order aspects of attention, such as attention shifting, are typically assessed by asking subjects to switch attention to new stimulus dimensions, such as color or shape, as in the Dimensional Change Card Sort (DCCS, Frye et al., 1995).

Noterdaeme et al. (2001) compared the attention profiles of 19 participants with ASD (aged 7 to 21) to participants with DLD and typical development, matched for age, sex and non-verbal IQ. Although both ASD and DLD groups showed impaired executive functions (inhibition and attention shifting), only the DLD group displayed additional deficits in lower-order skills (sustained auditory attention and selective attention). In the same vein, Tye et al. (2014) compared children aged 8 to 13 with ASD and with Attention Deficit/Hyperactivity Disorder (ADHD) with IQ in the average range, on a flanker-cued continuous performance task. They showed that children with ADHD displayed deficits in low-order skills, such as attentional orienting, whereas those with ASD showed deficits in conflict monitoring and response preparation. Studies of sustained attention in children with ASD have conflicting results, with some documenting deficits (e.g., Chien et al., 2014, 2015; Vivanti et al., 2017) and others not (e.g., Garretson et al., 1990; Johnson et al., 2007). These differences could be explained by the heterogeneity of participant ages and nonverbal cognitive functioning. In a review of attention, inhibition and cognitive flexibility research in average-IQ children with ASD, Sanders et al. (2008) identified frequent deficits in orienting attention, inhibition and shifting, but apparently typical capacities in sustained attention. A meta-analysis conducted by Demetriou et al. (2018) also confirms the deficit in executive functions in ASD, notably in mental flexibility. The current study measures the impact of these cognitive functions in ASD on linguistic abilities, and in particular how WM may impact syntax.

## Working Memory and Syntax

Executive functions are linked to syntactic capacities, in typically-developing children (e.g., Finney et al., 2014; Ibbotson & Kearvell-White, 2015; Viterbori et al., 2012; White et al., 2017), in children who present either syntactic difficulties, such as children with DLD (Ellis Weismer & Thordardottir, 2002; Im-Bolter et al., 2006; Montgomery, 2008; Montgomery et al., 2009), and in executive dysfunction such as ADHD (Stanford & Delage, 2020).

While many of these studies focused primarily on selective attention and attention shifting, WM (and verbal WM in particular) is also linked to syntax. For example, Adams and Gathercole (2000) and Willis and Gathercole (2001) reported that children aged 3–5 years with lower phonological loop abilities (evaluated with forward digit span and nonword repetition) produced significantly fewer correct complex sentences compared to children with better WM performance. De Abreu et al. (2011) compared the role of simple and complex spans on the language performance of typically-developing 5-year-olds. They reported that simple spans (assessed via forward digit span and nonword repetition) were more closely related to lexical abilities, and complex spans (assessed via backward digit span and counting span) were most closely related to syntactic comprehension. Montgomery et al. (2008) compared the role of simple spans (assessed via nonword repetition) and complex spans (assessed via listening span) on complex sentence comprehension in children aged 6 to 12, and found that complex (but not simple) spans explained a significant part of the variance (30%) in syntactic comprehension scores.

Delage and Frauenfelder (2019) conducted a detailed assessment of the relationship between simple and complex components of WM and complex syntax (assessed in comprehension, repetition and spontaneous production) in 48 monolingual French-speaking children aged 5 to 12. They found a strong predictive relationship between WM and syntax, with no effect of non-verbal IQ. This relationship was even more pronounced for more complex syntactic structures, such as relative clauses with multiple versus single levels of embedding and object relatives versus subject relatives. Complex spans (particularly counting span) explained the largest part of the variance in the production of embedded utterances in spontaneous speech, whereas the production of simple sentences (without any embedding) was not predicted by WM. These results highlight the link between WM and complex syntax: “Hence, it seems that processing complex sentences, even in a natural contact, depends on WM capacities, which is not the case for simple sentences that contain fewer syntactic operations, such as the multiplicity of internal and external merges” (Delage & Frauenfelder, 2019, p. 165).

## Working Memory and Syntax in DLD

Numerous studies have examined the link between WM and syntax in children with atypical development. Stanford (2020) reported strong correlations between WM (assessed via forward and digit span) and expressive syntax in 20 French-speaking children with ADHD aged 6 to 10. Montgomery and Evans (2009) showed a significant link between WM and comprehension of complex sentences in 24 children with DLD aged 6 to 12, a link which was stronger than that in the typically-developing comparison group. Frizelle and Fletcher (2015) identified close relationships between WM and complex sentence repetition in 35 children with DLD aged 6–7, echoing findings from Riches et al. (2010) in 14 adolescents with DLD aged 14 to 16. Testing a clinical marker of DLD, namely the 3rd person accusative clitic in French (e.g., il **le** lave ‘he’s washing him/it’), Durrleman and Delage (2016) found a correlation between this grammatical marker and a measure of complex span (backward digit span) in 22 participants with DLD aged 5 to 16. This study also probed elicited production of first-person accusative clitics (e.g., il **me** lave ‘he is washing me’), which do not require the morphological marking of gender and number,^2^ and found no correlation with WM.

Delage and Frauenfelder (2020) evaluated the performance on complex syntax and WM of 28 French-speaking children with DLD aged 5 to 14, who completed three simple-span tasks (forward digit span, serial order memory and nonword repetition) and three complex-span tasks (backward digit span, counting span and running span). Results highlighted the severe deficits of children with DLD in both syntax and WM; further, deficits were largely uncorrelated with age (one single correlation on 11 measures), in spite of the large age range of the group, whereas such correlations were present in 28 aged-matched peers (correlations on eight of 11 measures). WM skills predicted the comprehension and repetition of complex sentences, controlling for non-verbal IQ, and simple spans (especially, the serial component of verbal short-term memory) predicted syntactic measures in spontaneous language.

Given that WM limitations in children with DLD predicted their (deficient) complex syntax abilities, WM training in this population was the logical continuation of this research area. Our previous work (Delage et al., 2020, 2021; Stanford et al., 2019) evaluated the effects of WM training on syntactic abilities of children with DLD via a novel WM training program, *Magic Memory* (Delage et al., 2017). This program included exercises targeting the aspects of WM most predictive of syntax (Delage & Frauenfelder, 2019, 2020). 32 children with DLD (aged 6–12) received 8 weeks of *Magic Memory* training; an age-matched DLD comparison group completed an alternative training. Findings revealed both direct benefits on untrained WM tasks and indirect benefits on expressive syntax. Specifically, the WM-training group had more accurate elicited production of 3rd person accusative clitics (Stanford et al., 2019) and repetition of complex sentences (Delage et al., 2020, 2021). The active comparison group showed no such improvements, suggesting the specificity of the observed effects of the target WM training.

## Working Memory and Syntax in ASD

In summary, language profiles in both ASD and DLD suggest difficulties with complex structures such as accusative clitics (Durrleman & Delage, 2016; Prevost et al., 2018), relative clauses (Riches et al., 2010; Silleresi et al., 2018), morphological marking (Modyanova et al., 2017; Roberts et al., 2004), *wh*-questions (Durrleman et al., 2016; Prévost et al., 2017; Zebib et al., 2013), passives (Ambridge et al., 2021; Durrleman et al., 2017) and embedded clauses (Durrleman et al., 2019; Silleresi et al., 2018). WM deficits are also attested in both ASD and DLD (e.g., Habib et al., 2019 for ASD or Kapa & Plante, 2015 for DLD). As in DLD, there appears to be a specific relationship between WM and syntax in children with ASD; those children who have the most difficulty with language tasks also have more difficulty with phonological WM (Kjelgaard & Tager-Flusberg, 2001). Hill et al. (2015) distinguished between ASD children with and without comorbid language deficits; children in the former group had more severe WM deficits than the latter. In Durrleman and Delage (2016), 21 French-speaking participants with ASD aged 5 to 16 and 22 age-matched children with DLD completed standardized measures of expressive grammar and the production of a clinical marker of DLD in French, i.e. accusative clitics, alongside measures of verbal WM (forward and backward digit spans). Both groups showed impaired WM skills, which also correlated with production of 3rd person accusative clitics; non-verbal IQ did not correlate with any measure of syntax. Weismer et al. (2017) compared children with ASD and DLD (30 per group, mean age 10 years) in an N-back WM task and a complex grammatical judgement task, and found performance to be highly correlated in both groups. Similarly, Schuh and Eigsti (2012) demonstrated the presence of a strong relation between phonological WM (assessed via nonword repetition) and syntactic performance (assessed via the syntactic scale of CELF-4, Semel et al., 2003) in individuals with ASD and average cognitive abilities, ages 9–17. Riches et al. (2010) reported correlations for performance on complex sentence repetition, nonword repetition, and backward digit span in 16 adolescents with ASD plus language impairment, aged 14 to 15. In sum, while fewer studies have investigated this issue, there is robust evidence linking WM and syntax in ASD. As in DLD, WM limitations in ASD likely impact the acquisition of complex syntax, and improving WM capacities via a dedicated training program could free up cognitive resources to deal more effectively with syntax. The current study addresses this hypothesis.

## Research Questions

The empirical data presented above, particularly our training studies in DLD (Delage et al., 2020, 2021; Stanford et al., 2019), suggest that specific WM training could have a positive impact on the mastery of complex syntax in children with ASD. We extended the methods of the DLD WM training study to a population of children with ASD, using the *Magic Memory* (Delage et al., 2017) training program. We predicted (1) a significant increase in WM scores following training (**direct effects on WM**); and (2) a significant improvement in complex syntax (**transfer effects on syntax**). This study did not include an active control group of participants receiving an alternative training regimen, since our previous studies with children with DLD or typical development (Delage et al., 2020, 2021; Stanford et al., 2019) found no improvement in WM or syntax for participants receiving an alternative training program focusing on academic skills. There was no obvious reason to anticipate different results with a second active control group of ASD participants; thus, we focused our recruitment efforts on children to be included in the target (WM) training program.

The current study also included attentional measures, in light of the observation that WM is closely linked to the attentional system (Garon et al., 2008; Majerus et al., 2009; Veer et al., 2017) and children with ASD are known to experience difficulties in executive functions (Demetriou et al., 2018). Given that intensive training might have a specific impact on attention, we predicted (3) an improved performance in attentional measures after training (**transfer effects on attention**). Finally, participants completed a long-term follow-up three months after training. We predicted that 4) potential effects on WM, syntax and attention would be maintained after three months (**long-term effects**).

## Methods

**Fig. 1:**
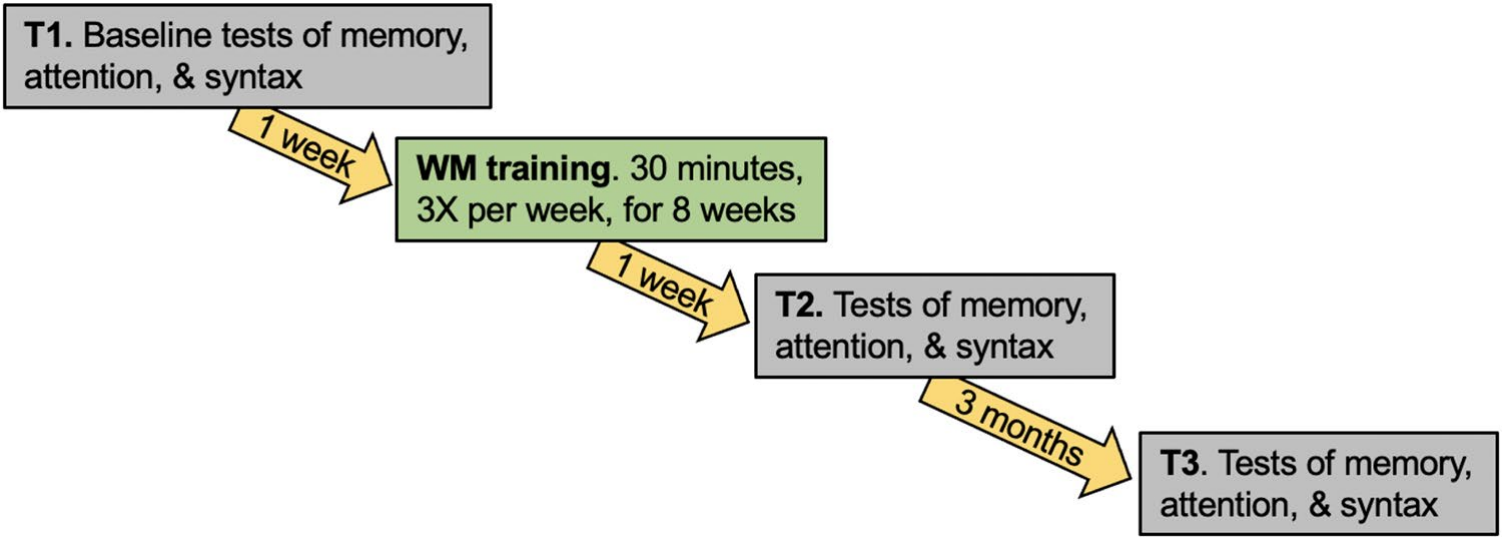
Timeline of study activities

For all participants, the study procedures were identical, see Fig. 1, and follow the procedures used in our previous studies in children with TD and DLD of the same age (Delage et al., 2020, 2021; Stanford et al., 2019). The training was delivered on iPad (that we provided), with frequent positive feedback (encouragements, rewards, playful animations) to boost motivation. Training sessions were carried out by caregivers at home under the supervision of speech-language therapy graduate students. These graduate students contacted parents on a weekly basis to ensure that the training program was being appropriately followed and visited participants two to three times a month to track the progress of the training regime. The graduate students also performed the different pre- and posttests and the same student always worked with the same child throughout the study. To ensure the fidelity of the analyses, all tests were recorded and the transcripts and scores were checked by two experts in the field, authors 1 and 3, respectively.

Baseline (**T1**) abilities were established via pre-tests 1 week prior to the first training session, comprising two 45-min sessions to assess memory, syntactic and attentional skills. Participants then completed the 8-week intensive training regimen of three 30-min sessions per week for a total of 12 h. Tests were completed again one week after training (**T2**) with exactly the same structures but different items, matched in frequency, length and complexity, to avoid practice effects. There were two different test versions, A and B, and administration order was counterbalanced such that half of the participants completed version A pre-tests and version B post-tests, and the other half completed the opposite order. Post-tests (**T3**) were administered three months after T2, to assess the long-term stability of effects. Participants completed the same version (A or B) at T3 that they had completed at T1, five to six months earlier. As indicated on Fig. 1, the whole process lasted 5 months and 2 weeks. All participants completed the entire set of tests at each timepoint (T1, T2 and T3). All test sessions were of equivalent duration, lasting approximately 90 min.

### Participants

We recruited 30 French-speaking children (3 girls) with ASD ages 5 to 11 (*M* = 8;8, *SD* = 1;8) from speech-language centers and specialized schools in the Geneva and Paris regions, which we had contacted directly to present the study and its clinical objectives. This age range was chosen since it corresponds to the ages of children for whom relationships between WM and complex syntax had previously been identified (Delage & Frauenfelder, 2019, 2020; Durrleman & Delage, 2016). We also chose structures known to be fairly well mastered from the age of 6 in typical development, for producing accusative clitics (Delage et al., 2016; Zesiger et al., 2010) and root questions (Jakubowicz, 2011), for repeating relative clauses (Frizelle & Fletcher, 2014; Frizelle et al., 2017) as well as for comprehending complement sentences such as those tested here (Durrleman et al., 2019). Moreover, in our previous training studies using the same materials and protocols, we reported that TD children (aged 6–12) trained on WM showed improved WM performance thanks to the WM training, while their syntactic scores did not improve, given that performance approached ceiling both prior to and following training (Delage et al., 2021; Stanford et al., 2019).

Inclusion criteria were: (i) meeting DSM-5 (American Psychiatric Association, 2013) criteria for ASD according to the Autism Diagnostic Observation Schedule, ADOS (Lord et al., 2002; translated by Rogé et al., 2015) and/or the ADI-R (Rutter et al., 2003a, 2003b; translated by Rogé et al., 2011). The diagnosis of ASD was given by a child psychiatrist or psychologist with ASD expertise; (ii) low scores (≤ 1 SD below the mean) on standardized tests of syntax and complex WM; and (iii) French as a first language. For all participants, we used the French adaptation of the Childhood Autism Rating Scale (CARS, Schopler et al., 1980; translated by Rogé, 1989) to assess the severity of autistic symptomatology, see individual scores in Appendix A. Non-verbal reasoning was assessed using Raven’s progressive matrices (Raven & Court, 1998). Five children were included in the study despite a low Raven’s score (< 2 SD) because the examiners determined that these children had sufficiently strong comprehension and attention to participate. In addition, six children were simultaneous bilinguals,^3^ two had comorbid ADHD and two others followed specialized education (see Appendix A). Table 1 presents demographic data and T1 standardized test scores for measures of non-verbal reasoning (Raven’s score), expressive grammar (a sub-test of the BILO-3C battery, Khomsi et al., 2007) and WM (Evaluation of Working Memory Test, Boutard & Gatignol, 2015). Two composite simple- and complex-span scores were calculated for the WM standardized tasks.

**Table 1:** Standardized assessments of non-verbal reasoning, expressive grammar and working memory

	Chronological age	Non-verbal reasoning	Expressive grammar	WM Simple span	WM Complex span
M (SD)	8;8 (1;8)	− 0.6 (1.4)	− 3.7 (2.1)	− 1.2 (1.2)	− 2.5 (1)
Range	5;11–11;10	− 4.2–1.7	− 8.9 to − 1	− 4.1–1.1	− 5 to − 1

WM was assessed using the Evaluation of Working Memory Test (Boutard & Gatignol, 2015). Nonverbal IQ was estimated using Raven’s Progressive Matrices. Expressive grammar was assessed using the BILO-3C (Khomsi et al., 2007). Data are shown as Z-scores, calculated with respect to the normative scores as reported in test manuals

Nonverbal skills were age-typical overall; 14 children had above-average Raven’s scores. In expressive syntax, all children obtained low scores, with 20 scoring more than 2 SD below the mean. All children obtained low complex-span WM scores (> 1 SD below the mean for their age); simple span scores were more variable, and the difference between simple and complex spans was significant, *t*(58) = 4.52, *p* < 0.001. Appendix A provides these results in detail.

This study was approved by the Ethics Committee of the Faculty of Psychology and Educational Sciences of the University of Geneva as well as from the Geneva Cantonal Ethics Commission and was also declared at ‘La Commission Nationale de l’Informatique et des Libertés (CNIL)’ in France. Parents provided written informed consent for participation.

### Pre- and Post-training Tests: Working Memory

Before and after training, children completed tests assessing WM with three simple span tasks and two complex span tasks, see Table 2.

**Table 2:** Pre- and post-training tests of working memory

Task	Description	Scoring
Simple span tasks		
Forward digit recall (WISC IV, Wechsler, 2005)	The experimenter says aloud a series of digits increasing in length from 2 to 9; participants have to immediately repeat them aloud in the same order. Testing is discontinued when participants fail two trials in a row.	Number of correctly repeated sequences
Nonword repetition (BELEC, Mousty et al., 1994)	The experimenter says aloud a non-word, which the participant must repeat immediately. Words increase in length from 1–5 syllables and in phonological complexity (with Consonant–Vowel and Consonant–Vowel–Consonant structures), such as *moga*, *juséga* or *kragrinblan*. There is no stop criterion. A trial is marked as incorrect when participants omit, add or misorder one phoneme.	Number of correctly repeated syllables
Serial order word span (Majerus, 2008)	This task tests the ability to retain serial order information. The experimenter says aloud sequences of familiar animal names along with the order in which these animals finished in a race. Participants must place animal cards in the order in which they finished; thus, they must store and recall the serial order of items but not the item names. Sequence length increases from two to seven. Testing is discontinued when participants fail two trials in a row.	Number of items retrieved in the correct order
Complex span tasks		
Backward digit recall (WISC IV, Weschler, 2005)	The experimenter says aloud a series of digits increasing in length from 2 to 9; participants have to immediately repeat them aloud in reversed order. Testing is discontinued when participants fail two trials in a row.	Number of correctly repeated sequences
Counting span (Case et al., 1982)	Participants are asked to count the number of blue dots on each page; after completed from 1 to n pages, and signaled by a smiley face, they are asked to recall the tallies from each of the previous pages in the correct order. The number of pages increases until a stop criterion (two failures in a row) was reached. Testing only proceeds if the child is able to count collections of up to 11 items.	Number of digits retrieved in the correct order

### Pre- and Post-training Tests: Syntax

Before and after training, children completed three measures assessing syntactic knowledge, shown in Table 3. Each measure included two paired versions (A and B) for counterbalancing. To adapt the measures, we split the items in the initial tasks into two, leaving two versions that were matched on (1) lexical frequency and (2) the type of syntactic structure targeted.

**Table 3:** Pre- and post-training tests of syntax

	Tasks	Item structure (number of trials)
Elicited production	Root questions	N subject and object questions (/12) N *wh*-fronted object questions (/9)
	Accusative clitics	N correct clitics (/12)
Repetition	Complex sentence repetition	N correct syllables (/210) N respected target structure (/15) N respected degree of embedding (/15)
Comprehension	Complement sentences	N correct responses (/12)

#### Elicited Production of Root Questions

In this task, adapted from Jakubowicz (2006), the experimenter showed the child a picture in which a character performs an action with part of the image hidden. The child was prompted to ask the character a question about the hidden part (e.g., “Look, there is a rabbit pushing someone, but we don’t know who. Ask him”). The target responses consisted of subject (N = 3) or object questions (N = 9). For object questions, the child could formulate questions with a *wh*-fronted object (1 and 2) or *wh*-in situ questions (3), which are less grammatically complex but very frequent in spoken French.

1. “**Qui** pousses-tu?” (‘Who push you’).
2. “**Qui** tu pousses?” (‘Who you push’).
3. “Tu pousses **qui**?” (‘You push who’).

#### Elicited Production of Accusative Clitics

In this task adapted from Tuller et al. (2011) and Delage et al. (2016), the child saw images on a computer and then answered questions requiring the use of a 3^rd^ person accusative clitic (e.g. *what is the doctor doing with the boy?* Expected response: “*il ****le**** pèse*” ‘he’s weighing him’). Each version included 12 trials. Responses typically contained (i) the target accusative clitic, (ii) a lexical unit (il pèse **le garçon** ‘he’s weighing the boy’); this response is grammatical but infelicitous as it unnecessarily repeats the full lexical unit; (iii) a clitic with a gender error (ex: *il **la** pèse ‘he’s weighing her’), or (iv) an omitted clitic (*il pèse ‘he’s weighing’).

#### Complex Sentence Repetition

To test the ability to repeat syntactically complex sentences, we used a sentence repetition task created by Delage and Frauenfelder (2019) that required participants to immediately repeat sentences read to them by the experimenter. The task comprised 23 sentences, all 14 syllables long: Eight syntactically simple sentences (without any embedding) and 15 syntactically complex sentences. The complex sentences varied in target structure (5 subject and 10 object relatives) and in the number of embeddings (one, two or three). Target structures thus contained either a subject (as in 4) or an object relative (5), and an expected degree of embedding; examples (4) and (5) have one degree of embedding and (6) and (7), respectively, have two and three levels of embedding. Appendix B presents the entire set of sentences for the two versions (A and B), which were matched for length, syntactic structure and frequency.

(4) La maîtresse voit le garçon [qui lit un livre sur Noël]
  ‘The teacher sees the boy who is reading a book about Christmas’
(5) C’est un chat [que caressent tous les enfants après l’école]
  ‘It’s a cat that all of the children pet after school’
(6) Je crois [que la fille préfère le chien [qu’elle a colorié]]
  ‘I think that the girl prefers the dog that she colored’
(7) Il pense [qu’elle dit [que le garçon déteste la fille [qui pleure]]]
  ‘He thinks that she says that the boy hates the girl who is crying’

As the task progressed, the structures the children were asked to repeat became increasingly more complex. Scoring for the sentence repetition task considered (i) the number of syllables which were correctly repeated, disregarding possible mispronunciations; (ii) inclusion of the target structure (e.g. an object relative), and (iii) inclusion of the expected degree of embedding (one, two or three). Considerations (ii) and (iii) focused only on syntactic properties. Thus, if the response included the correct structure and/or the expected level of embedding, it was scored as correct even if the response included an incorrect word (e.g., if a child said “the woman” instead of “the teacher”). Responses were digitally recorded. Trained research assistants transcribed and coded all responses, which were reviewed in full by the first and the third authors.

#### Comprehension of Complement Sentences

Complementation skills were assessed using a task adapted from De Villiers and Pyers (2002). Children were presented with 12 scenes in which a character reported an event to another. Half of the items included an accurate reporting of the event, and half involved a mistaken or false belief. Children were required to recall the content of the (true or false) complement. For example, children heard: “The girl asks Dad what Mom is doing and Dad answers that Mom is working.” For true complements, the story continued: “Look, Mom is really working” while for false complements, children heard: “But look, Mom is actually taking a nap in the office.” In both instances, the test question was: “What does Dad say that Mom is doing?” (while pointing to the first picture); see Appendix C. The child had to point to the picture representing the content of the complement initially heard (i.e., depicting Mom working).

### Pre- and Post-training Tests: Attention

#### Selective Attention

Visual selective attention capacity was assessed via the Sky Search task (TEA-ch, Manly et al., 2006), which required participants to identify and circle pairs of identical spaceships from a page of visually-similar stimuli while ignoring distractors. Forty-nine spaceships were displayed and 20 were identical pairs. Speed (in milliseconds) and accuracy (number of correct targets) were the measures of interest.

#### Processing Speed and Inhibition

Performance on the Opposite Worlds task (TEA-ch, Manly et al., 2006), which was made up of two conditions, served as an indicator of attentional control/shifting. In the Same World condition, which measured processing speed, participants were presented visually with a path along which the digits 1 and 2 were scattered. The participants were asked to follow the path with their finger, naming each digit out loud (i.e. saying “one” each time they saw the digit 1 and “two” each time they saw the digit 2). In the Opposite World condition, which measured inhibition and response modification, the participants were instructed to say “one” when they saw a printed “2” and “two” when they saw a printed “1”. Errors resulted in a time penalty as participants were prevented from proceeding along the path to the subsequent digit until the error had been corrected. In total, the participants saw two worlds with Same World rules and two worlds with Opposite World rules that were presented in the following order: Same World 1—Opposite World 1—Opposite World 2—Same World 2. Scores were calculated as the average time to complete the Same World and Opposite Worlds conditions.

### Working Memory Training Program

The WM training was delivered via iPad using the same *Magic Memory* program used in our previous training studies (Delage et al., 2020, 2021; Stanford et al., 2019). In this adaptive program, the level of difficulty increased as a function of a child’s progress. Five different activities, detailed in Appendix D, were presented in random order. All participants followed the same activities (five activities at each training session), with identical rhythm trials (5 min per activity). Activities targeted serial-order memory, WM updating and dual-task processing. In the serial-order memory activity, the child heard a series of familiar monosyllabic words and used a finger to drag the corresponding images into a train car in the order of presentation. The WM updating activity was an *n*-back task in which participants clicked an object (on the screen) if it appeared one, two or three trials previously. In the dual-task processing activity, the child had to retain an ordered list of familiar auditory stimuli (such as household objects, animal noises or musical instruments) while simultaneously performing a secondary task (a visual comparison of quantity task).

## Results

After verifying that variables met standard assumptions of normality and heterogeneity, we checked for correlations between WM scores and syntax, as well as correlations of WM/syntax to clinical variables (i.e., age, non-verbal reasoning and autistic symptomatology). As a first step in the analyses, we created composite variables for WM, syntax, and attention measures (see “preliminary analyses” for details of these calculations). These composites were subjected to repeated-measures ANOVAS, to test for a main effect of the intervention (T1, T2, T3) on WM (direct effects) and syntax and attention (transfer effects). Those composite measures that showed significant change over time were further subjected to repeated-measures ANOVAs to compare performance from T1 to T2 (intervention effects) and from T1 to T3 (long-term effects); we also conducted exploratory analyses of individual WM, syntax, and attention measures, to identify specific domains where the intervention had the greatest impact. Finally, additional analyses probed clinical and cognitive predictors of changes in WM and syntactic ability.

### Preliminary Analyses

Before exploring the effects of WM training, we ascertained whether WM and syntax were correlated at T1-Baseline, as in previous studies (Durrleman & Delage, 2016; Schuh & Eigsti, 2012). We calculated unit-weighted standardized (*Z*-score) composite scores for simple and complex spans; each measure contributed equally to the composite. The simple span composite was the average of forward digit recall and serial order word span *Z*-scores; these individual measures were highly correlated, *r*(30) = 0.60, *p* < 0.001. Nonword repetition (phonological WM) was excluded from this composite, as it was uncorrelated with serial order word span, *r*(30) = 0.29, *p* = 0.11. The complex span composite was the average of backward digit recall and counting span z-scores, which were also highly correlated, *r*(30) = 0.47, *p* = 0.006. Results showed that the complex but not simple span composite correlated with all syntactic measures; see Table 4. We also tested correlations of syntax and WM with age, non-verbal reasoning (Raven’s matrices) and autistic symptomatology (CARS score); none were significant. The syntax composite was calculated as the average of the *Z*-scores of (1) Elicited production of root questions, (2) Elicited production of accusative clitics, (3) Sentence repetition, and (4) Comprehension of complement sentences. The attention composite was calculated as the average of the *Z*-scores of (1) Sky Search and (2) Opposite Worlds tasks.

**Table 4:** Pearson correlations between composite WM scores and measures of syntax

	Elicited production		Complex sentence repetition		Complement sentence comprehension
	Root question accuracy	Clitic accuracy	N correct syllables		N correct responses
Simple span composite	0.31	0.29	0.34(±)		0.26
Complex span composite	0.42*	0.37*	0.52**		0.42*

Notations in bold indicate statistically significant results

**p < .01; *p < .05; (±) marginally significant (*p* < .07); df = 30

### Improving Working Memory: Direct Effects

To test whether WM training led to significant improvements in WM performance, we performed an initial repeated-measures ANOVA on T1, T2 and T3 scores. Both the simple WM composite scores, *F*(1, 29) = 27.89, *p* < 0.001, Cohen’s *f* = 0.93, and complex WM composite scores, *F*(1, 28) = 185.89, *p* < 0.001, Cohen’s *f* = 2.48, showed significant main effects of training with large effect sizes. As such, we performed follow-up analyses to compare T1 with T2, for each of the WM measures; scores and statistics are presented in Table 5. Results indicated a significant effect of training, with a medium to large effect size, for all WM tasks, with improvements from T1 to T2.

**Table 5:** Repeated-measures ANOVA on working memory measures

	Pretest T1	Posttest T2	T1-T2 Effect of Time	Posttest T3	T1-T2-T3 Effect of Time	Post hoc HSD		
	M (SD)	M (SD)		M (SD)		T1-T2	T1-T3	T2-T3
N participants	30			26				
Forward digit recall (max = 16)	4.7 (1.6)	5.8 (1.8)	***F*(1, 29) = 12.73,** ***p* < .001, η**^**2**^** = .31**	5.4 (1.5)	***F*(2, 50) = 8.56,** ***p* < .001, η**^**2**^** = .25**	***p* < .001**	***p* = .02**	*p* = .5
Nonword repetition (max = 80)	40.9 (13.6)	46.3 (9)	***F(1, 29)* = *6.84,*** ***p* = *.014,* η**^**2**^** = .19**	45.1 (12.9)	***F*(2, 50) = 3.91,** ***p* = .02, η**^**2**^** = .13**	*p* = .09	***p* = .03**	*p* = .9
Serial order word span (max = 81)	19.2 (11.3)	30.3 (10.2)	***F(1, 29)* = *49.92,*** ***p* < *001,* η**^**2**^** = .60**	27.5 (12.5)	***F*(2, 50) = 17.03,** ***p* < .001, η**^**2**^** = .40**	***p* < .001**	***p* < .001**	*p* = .4
Backward digit recall (max = 16)	4.1 (2.3)	5.4 (1.9)	***F(1, 29)* = *16.42,*** ***p* < *001,* η**^**2**^** = .36**	4.5 (2.2)	***F*(2, 50) = 9.92,** ***p* < .001, η**^**2**^** = .28**	***p* < .001**	*p* = .4	***p* = .01**
Counting span (max = 81)	8.6 (9.7)	12.6 (13.9)	***F(1, 29)* = *11.10,*** ***p* = *002,* η**^**2**^** = .28**	11 (13.9)	***F*(2, 50) = 5.06,** ***p* < .01, η**^**2**^** = .17**	***p* = .008**	*p* = .1	*p* = .5

Notations in bold indicate statistically significant results

T3 data include only 26 out of 30 participants, four children having not been retested due to the coronavirus COVID-19 pandemic

### Improving Syntax: Transfer effects

Repeated-measures ANOVAs tested whether training effects transferred to syntax. An initial repeated-measures ANOVA on T1, T2 and T3 composite syntax scores revealed a significant effect of training with large effect size, *F*(1, 26) = 98.16, *p* < 0.001, Cohen’s* f* = 1.86. As such, we performed follow-up analyses to compare T1 with T2, for each of the syntax assessments; scores and statistics are presented in Table 6. Each of the measures showed a mean increase in accuracy, though not all changes were statistically meaningful. Of the four elicited syntactic production measures, root questions significantly improved between T1 and T2; of these root questions, only object questions, including *wh*-in situ items, showed significant change, *t*(29) = − 2.2, *p* = 0.03, *d* = 0.4. There was no significant change in *wh*-fronted questions or object clitic productions. For complex sentence repetition, the number of correctly repeated syllables significantly improved from T1 to T2, with a medium-to-large effect. This result cannot only be explained by better memory, because repetition accuracy for simple sentences (which contained an identical number of syllables) showed no significant improvement, *t*(29) = − 1.2, *p* = 0.2. The percentage of sentences in which the degree of embedding was respected, independently of the lexical units, also improved. Finally, the children also showed a significant improvement in complement sentence comprehension between T1 and T2.

**Table 6:** Repeated-measures ANOVA on syntax and attention

		Pretest T1	Posttest T2	T1-T2 Effect of Time	Posttest T3	T1-T2-T3 Effect of Time	Post hoc HSD		
		M (SD)	M (SD)		M (SD)		T1-T2	T1-T3	T2-T3
N participants		30			26				
Elicited production	N correct root questions (max = 12)	6.8 (4.1)	7.9 (3.2)	***F*(1, 29) = 5.64,** ***p* = .02, *η***^***2***^ **= .16**	7.7 (3.9)	***F*(2, 50) = 5.95, *p* = .005, η**^**2**^** = .20**	***p* = .006**	***p* = .03**	*p* = .8
	N *wh*-fronted object questions (max = 9)	3.2 (2.6)	3.5 (2.8)	*F*(1, 29) = 0.41, *p* = .52					
	N correct clitics (max = 12)	2.8 (3.6)	3.2 (3.5)	*F*(1, 29) = 1.43, *p* = .24					
Complex sentence repetition	N correct syllables (max = 210)	119.1 (44.9)	138.1 (39.2)	***F*(1, 29) = 15.82, *p* < .001, *η***^***2***^ **= .35**	134.2 (35.1)	***F*(2, 50) = 11.5, *p* < .001, η**^**2**^** = .31**	***p* < .001**	***p* = .003**	*p* = .5
	N target structure (max = 15)	4.5 (3.8)	5.3 (4.3)	*F*(1, 29) = 2.27, *p* = .14					
	N degree of embedding (max = 15)	3.5 (3.5)	4.4 (3.7)	***F*(1, 29) = 4.55,** ***p* = .04, *η***^***2***^ **= .14**	4.2 (3.5)	*F*(2, 50) = 2.72, *p* = .07, η^2^ = .10	*p* = 0.08	*p* = .2	*p* = .9
Complement sentence comprehension	N correct responses (max = 12)	7.4 (2.4)	8.3 (2.5)	***F*(1, 29) = 9.13,** ***p* = .005, *η***^***2***^ **= .24**	7.9 (2.8)	*F*(2, 50) = 2.07, *p* = .14, η^2^ = .08	*p* = .1	*p* = .5	*p* = .7
Selective attention	Speed (ms)	9.2 (4.2)	7.3 (4.3)	***F*(1, 29) = 6.04,** ***p* = .02, *η***^***2***^ **= .17**	7.5 (3.7)	***F*(2, 50) = 6.05, *p* = .004, η**^**2**^** = .19**	***p* = .005**	***p* = .03**	*p* = .8
	Accuracy (max = 20)	15.9 (3.9)	14.8 (4.6)	*F*(1, 29) = 1.98, *p* = .17					
Processing speed	Speed (ms)	34.4 (11.9)	30.3 (9.3)	***F*(1, 29) = 9.57,** ***p* = .004, *η***^***2***^ **= .25**	29.9 (7.6)	***F*(2, 50) = 8.45, *p* < .001, η**^**2**^** = .25**	***p* = .008**	***p* < .001**	*p* = .7
Attention shifting	Speed (ms)	48.2 (20.4)	39.4 (15.2)	***F*(1, 29) = 17.21, *p* < .001, *η***^***2***^ **= .37**	36.5 (10.2)	***F*(2, 50) = 16, *p* < .001, η**^**2**^** = .39**	***p* < .001**	***p* < .001**	*p* = .7

Notations in bold indicate statistically significant results

T3 data include only 26 out of 30 participants, four children having not been retested due to the coronavirus COVID-19 pandemic

### Improving Attentional Skills: Transfer Effects

To test the hypothesis that WM training would boost selective attention, processing speed and attention shifting, we conducted a third repeated-measures ANOVA on the composite T1, T2 and T3 attention scores; this revealed a significant effect of training, with a large effect size, *F*(1, 25) = 442.31, *p* < 0.001, Cohen’s* f* = 4.04. As such, we employed a series of repeated-measures ANOVAs, detailed in Table 6, to test specific effects. All measures showed a significant decrease in reaction time between T1 and T2, revealing faster processing. The largest effect was observed for the most demanding task, the Opposite World task, which required the participant to inhibit a prepotent response.

### Long-Term Effects

**Fig. 2:**
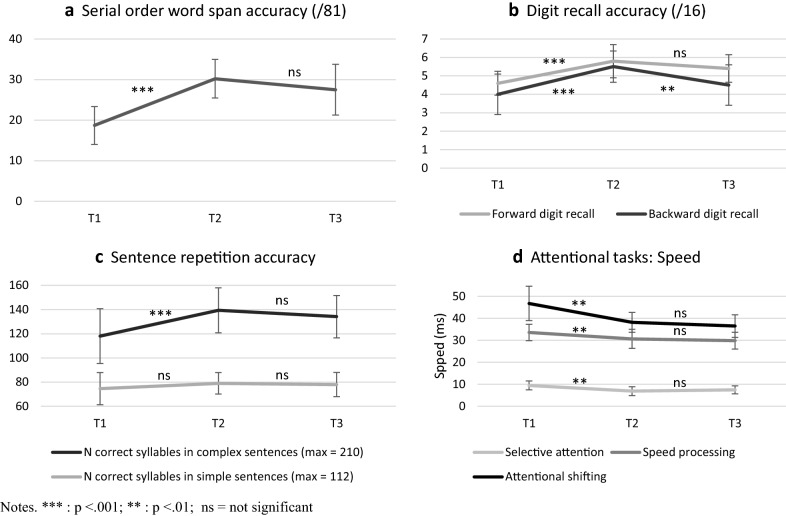
T1, T2 and T3 performance for serial order word span and digit recall tasks, sentence repetition and attentional tasks

Of the original sample of 30, 26 children with ASD were retested at T3, three months after the posttest. Repeated measures ANOVAs with time (T1, T2, T3) as a within-subjects factor revealed significant main effects of time were observed for each of the WM tasks; see Table 5. Post-hoc Tukey’s HSD tests explored the periods for which significant change occurred. Performance at T3 was significantly better than T1 for the three simple-spans tasks (forward digit span, nonword repetition and serial order word span), but not for the two complex-span tasks. Comparisons of T2 and T3 showed no significant changes except in backward digit recall which showed a significant decrease (e.g., worse performance). Figure 2 illustrates these results for the serial order word span (2a) and forward digit recall (2b), which followed the predicted pattern of significant improvements between T1 and T2 and between T1 and T3 and no change between T2 and T3. Performance on the backward digit recall task (2b), however, decreased significantly from T2 to T3.

The long-term stability of transfer effects was calculated for those measures of syntax and attention for which a significant T1-T2 increase was observed. As indicated in Table 6, the gains in syntactic abilities observed at T2 were still present at T3, with no significant decrement in performance. This pattern held for both syntactic measures (elicited production of root questions, sentence repetition, see Fig. 2c) and for each of the three attentional tasks (2d).

### Further Analyses

The results revealed a significant impact of WM training on WM itself (direct effects) and on attention and syntax (transfer effects); improvements in the latter were in the moderate range. Given the heterogenous nature of ASD, it was important to test whether improvements were observed for all participants. We calculated gains for the primary measures^4^ by subtracting the T2 results from T1 results for each child. Appendix E presents these measures of gains for each child as well as group means for each measure. As expected, improvements were extremely variable with some children making progress on all the tasks (such as participants 4 or 30) and others showing no real improvement on any task (such as participants 17 or 20). We tested whether age, non-verbal reasoning, or autistic symptomatology predicted gains, using correlational analyses; none were significant, all *p*’s > 0.10.

## Discussion

Our study explored the effects of an intensive WM training program, *Magic Memory* (Delage et al., 2017), on WM, syntax and attention, for 30 French-speaking children with ASD aged 5;11 to 11;10. We expected similar results in ASD as those previously reported for similarly-aged children with DLD, namely, direct effects on WM as well as transfer effects on expressive syntax (Delage et al., 2020, 2021; Stanford et al., 2019). In the present work, we aimed to replicate and expand upon our previous studies with participants with DLD, and related studies reporting WM/syntax links in ASD (Durrleman & Delage, 2016; Riches et al., 2010; Schuh & Eigsti, 2012; Weismer et al., 2017). The current study employed measures used in previous training studies in DLD, along with several additional tasks:

i. An assessment of the comprehension of complement sentences;
ii. Tasks assessing selective attention, speeded processing and attentional shifting;
iii. Delayed post-tests three months after training, to test for long-term effects.

We predicted improved performance on the capacities directly trained, i.e. on WM, as well as on syntactic and attention domains not directly trained but hypothesized to be related to WM. We also predicted that these gains would be maintained three months after training.

### Links Between WM and Complex Syntax

Preliminary analyses confirmed the close relation between complex-span measures (i.e., complex WM) and all measures of syntax in our participants with ASD, replicating previous results (Durrleman & Delage, 2016; Riches et al., 2010; Weismer et al., 2017). The fact that simple spans did not appear to be linked to the same extent to syntactic capacities in our population suggests that the more executive component of WM plays a role in complex syntactic processing in ASD. This result echoes findings of Delage and Frauenfelder (2019) who reported that complex spans (but not simple ones) predicted measures of syntactic complexity in spontaneous language samples of 48 TD children; this pattern was reversed in DLD (Delage & Frauenfelder, 2020). This larger pattern of results suggests that the WM deficits in DLD reflect reduced phonological storage (see also Alt, 2011; Gathercole & Baddeley, 1990), whereas the WM deficits in ASD reflect executive dysfunction, notably in mental flexibility (Demetriou et al., 2018). The current results are consistent with this hypothesis, as our participants with ASD had significantly better simple-span results (mean Z-score of − 1.2) relative to complex-span ones (mean Z-score of − 2.5). Their difficulties in processing complex sentences likely reflect inefficient or otherwise more impaired performance of the cognitive operations required for complex spans (i.e., reduced cognitive flexibility to cope with interference during verbal storage).

### Direct Effects of WM Training

The WM training was effective as it led to significant improvements in performance on all WM tasks. Admittedly, these tasks closely resembled the activities presented in the *Magic Memory* program, with distinct visual and verbal content and a distinct format (paper versus computerized). Nevertheless, such direct effects have not been consistently observed (Majerus, 2016; Melby-Lervag & Hulme, 2013) and it was encouraging to see that the children were able to transfer their skill from one format to another.

Effect sizes were impressive, with medium to large effects, with the exception of non-word repetition, for which improvements were significant with a small effect size. Speech-sound difficulties have been reported for subgroups of children with ASD (Kjelgaard & Tager-Flusberg, 2001; Wolk et al., 2016; Zebib et al., 2013), and such difficulties may have obscured the training effects. The serial-order-word-span task, in contrast, displayed the highest effect size. This task of putting animal cards on a podium in order is very similar to the training program’s task of putting pictures corresponding to familiar words into train cars in order, similarity that undoubtedly contributed to the large gains that participants displayed in this particular task. In addition, this serial memory task requires participants to remember the order of animals participating in the race, without necessarily retaining phonological representations of the animals’ names (Majerus, 2008; Majerus et al., 2006). Thus, this task focuses on WM while minimizing the influence of language, and of potentially degraded phonological representations. The finding that the task that least depends on phonological representations showed the most pronounced training effects is consistent with the aims of the training, which is meant to enhance “pure” memory processes (while also utilizing verbal material). These results suggest that this program will be effective for other conditions characterized by phonological deficits, including some forms of ASD and children with speech-sound disorders (Claessen & Leitão, 2012; Gathercole & Baddeley, 1990; Leonard, 2014; Zebib et al., 2013).

### Transfer Effects of WM Training on Syntax

All syntactic measures, both expressive and receptive, showed a mean increase in accuracy, with significant improvement in the production of root questions, and in the repetition and comprehension of complex sentences. Such transfer effects cannot be attributed to the material used in training, since the WM program presented only isolated words. Results of the sentence repetition in particular were striking, because they suggested significant increases at post-test for complex but not for simple sentences, though both types of sentences were matched in length. Similarly, independent of sentence length, participants showed improvement in producing complex sentential embedding.

Taken together, these exciting results suggest a meaningful transfer of WM improvement into the domain of complex expressive syntax, extending previous findings of training effects for children with DLD (Delage et al., 2021). Not all of the previous DLD results were replicated here; for example, there was no significant improvement in the production of accusative clitics (Stanford et al., 2019). Differences between response patterns in DLD and ASD likely reflect the nature of the tasks and the deficits specific to the autistic spectrum. For example, sentence repetition task involves limited social interaction; participants simply repeat the target sentence. In contrast, the clitic production task, which is understood by age four in typical development (Delage et al., 2016), requires more direct interaction with the experimenter, who asks questions (ex: *Look*!* What is the man doing with his car*? *Tell me!*) about pictured scenes. While the social demands are highly structured and relatively small, participants must listen and respond to the examiners; this could be a more difficult task for children with ASD (DSM-5, APA, 2013). However, the current results were not consistent with this hypothesis, as there was no relationship between syntax scores and autistic symptomatology (CARS scores). A more detailed assessment of the social communication skills, using the Children’s Communication Checklist (CCC-2, Bishop, 2003) for example, might have been more sensitive to individual differences in pragmatics.

Results also showed significant improvements in the comprehension of complement sentences. Receptive skills were not assessed in our training studies of children with DLD, who do display deficits in receptive syntax (Delage & Frauenfelder, 2020; Friedman & Novrogrodsky, 2004). Such receptive difficulties are widely reported in children with ASD, including deficits in the comprehension of complex *wh*-questions and relative clauses (Durrleman et al., 2016), passives (Durrleman et al., 2017), clitics (Terzi et al., 2014), and more globally on standardized measures of receptive syntax and morphology (Brynskov et al., 2017). Difficulties in comprehension of object relatives have been found to persist in young adults (Durrleman et al., 2015). In 2019, Durrleman et al. showed that comprehension of complement sentences can be improved in children with ASD, aged 5 to 11, through a brief training targeting sentential complements. In this study, WM training provided benefits in both cognitive and linguistic domains. Future work could compare the effects of training on complex syntax (as in Durrleman et al., 2019) to effects of WM training, in order to test whether effects are bidirectional. Such studies will illuminate language and cognitive deficits in this population.

### Transfer Effects of WM Training on Attention

In addition to the direct effect on WM capacities, WM training had an indirect effect on other attentional abilities, which is unsurprising given their theoretical overlap (Baddeley, 2003; Barrouillet & Camos, 2012; Engle, 2002; Majerus et al., 2009; Veer et al., 2017). Indeed, participants showed improved speed with no concomitant decrease in accuracy for the three aspects of attention: selective attention, processing speed and attention shifting. These gains suggest that WM training provides a cognitive “boost,” allowing children to process information more quickly, thereby freeing up processing resources. This change likely has a snowballing or cascading effect on syntactic processing as the reduction of cognitive limitations would influence the processing of computationally complex structures, such as those including embedding and/or a movement operation (Delage & Frauenfelder, 2019, 2020; Jakubowicz & Strik, 2008; Tuller et al., 2012). Attention limitations are also considered by Chomsky (2005) to impact the processing of syntax, and attention is seen as underlying the development of executive functions, such as WM and inhibition (Garon et al., 2008); given this foundation, we hypothesized that training in WM could lead to improvements on both attentional and syntactic measures.

Although previous WM training studies conducted with children with DLD have found the same direct and indirect effects (on syntax), these studies did not assess attentional abilities and were therefore unable to test for the transfer effects on attentional tasks that we obtained in the current study. Future studies should also evaluate these transfer effects in children with DLD. Similar effects would be expected, consistent with studies linking attentional resources to the processing of complex syntax in children with DLD (Montgomery et al., 2009; Stanford & Delage, 2020). Moreover, it would be informative to contrast effects of pure training of the attentional component (such as the TALI, Kirk et al., 2016) with effects in the current study, and to compare effects of both on syntax; this would reveal whether linguistic gains following intensive WM training can be attributed to WM improvements or to better functioning of the attentional system.

### Follow-Up

Almost all participants (26/30) were retested three months after training to assess long-term effects. Results showed that improvements were maintained in all WM tasks except backward digit recall, for which performance decreased from T2 to T3 (although T3 performance was still significantly better than at baseline). This task requires reversing the order of previously heard stimuli (digits). The training program was similar, but involving color names rather than digits. It is possible that the extensive overlap between tasks boosted the progress observed between T1 and T2, and that these gains were not maintained without practice (see Fig. 2b). Aside from this task, the overall results point in the direction of an encouragingly persistent and long-term improvement in WM performance, in contrast to previous WM training studies with healthy adults (Melby-Lervag & Hulme, 2013; Melby-Lervag et al., 2016). This suggests that WM training might be most effective with *children*, whose development is ongoing, or for individuals with specific WM deficits.

Long-term transfer effects were similarly positive, with improvements maintained on all measures showing T1 to T2 gains, whether in the areas of syntax or attentional skills. This result is particularly promising since we were unable to demonstrate maintenance of syntactic transfer effects in previous work, likely because only a small subset of children with DLD were retested (12 out of 32; Delage et al., 2021).

## Limitations

The present study did not include an active control group, which would have conclusively demonstrated that, without specific training, the performance in WM, syntax and attention would show significantly fewer improvements. As discussed above, we chose not to include such a group because our previous studies of children with DLD or TD revealed no benefit to WM or syntax from training focusing on academic skills (Delage et al., 2021; Stanford et al., 2019). While this is a significant limitation, we argue that the T2-T3 comparison provides a type of control, in line with ABAB treatment paradigms (e.g., Kratochwill et al., 2013). Such studies involve alternation between a baseline period (A) and a treatment period (B), sometimes including only a single subject. In such paradigms, the specific effects of the program should be visible following the training periods (B) and more discrete following the non-active (baseline) periods (A). This was precisely the pattern observed in the current study, in which the children’s performance did not improve between T2 and T3, suggesting that the progress observed from T1 to T2 was a function of the training program, rather than general maturation and development.

We also acknowledge that transfer effects from WM training onto syntax could be explained by other reasons than attentional resources and computational processing. Indeed, training sessions involve interactions between the child and the caregiver, as well as the experimenter. The increase in conversations with this diversity of interlocutors may also have helped to motivate children with ASD to engage in language tasks. In order to explore this aspect, it would have been useful to administer a questionnaire on the children’s social interactions, such as the Social Communication Questionnaire (Rutter et al., 2003a, 2003b), before and after the training. We leave this for future work. Other limitations stem from the fact that we did not evaluate the capacities of children to generalize their apparently improved syntactic skills to other daily life contexts, which would have been possible if we had carried out an analysis of spontaneous language. Complex syntax, assessed by such an analysis of spontaneous language samples, has already been shown to be strongly linked to WM in children with TD and DLD (Delage & Frauenfelder, 2019, 2020). We would thus expect that our training would also improve the children’s spontaneous syntax, yielding richer productions in conversational contexts. Moreover, it should be noted that our protocol was the same for all children, whereas the symptoms/traits of ASD are known to be heterogenous in nature. Adapting the material and the setting to the particularities of each child would certainly be useful in clinical practice, but this would not be suitable for a rigorous experimental design.

We should finally note that our statistical approach involved starting with an omnibus test of change in broad cognitive domains, with significant results to be followed by detailed exploratory tests of change in specific local task domains. While our results are compelling, and seem to be highly consistent across within-domain measures, this approach requires further replication given the relatively small sample size and the number of comparisons.

## Conclusion

Following WM training, our participants showed improvements not only in WM but also in distal domains such as speed of attentional processing and expressive and receptive syntax. Of course, these changes might reflect a general g-factor, such that the children with stronger cognitive abilities pay more attention in the training and show the greatest improvements. To address this possibility, future work could include a dynamic measure of cognitive functioning to directly examine the child’s response to learning (Camilleri & Law, 2007). Dynamic assessment of learning capacity provides a reliable and valid measure of general intellectual functioning and is a good predictor of future learning (Hessels & Hessels-Schlatter, 2010). Moreover, this type of assessment would be highly appropriate for children with emotional or personality challenges that could interfere with their performance (Tzuriel, 2001). A dynamic assessment could illuminate the inter-subject variability we observed in this study, with ASD participants who benefit more from WM training being those who show the better capacity to learn.

While they must be replicated, these exciting results provide impetus for further studies of WM interventions. Thereafter, the training materials should be available to children with WM or syntactic impairments, regardless of the proximal source of those impairments (e.g., DLD or ASD). Findings of this study provide further evidence in support of the efficacy of WM training in multiple domains, and fall within an ‘evidence-based practice’ framework, which emphasizes the role of research findings in clinical decision-making (Sackett et al., 2000).

## Supplementary Information

Below is the link to the electronic supplementary material.

Supplementary file1 (DOCX 220 KB)

## Footnotes

1 Diagnosis of DLD includes severe and persistent language disorders that interfere with daily functioning in the absence of a specific biomedical condition, such as Down Syndrome (Bishop et al., 2017).

2 See Delage et al., (2016) for further analyses.

3 We compared the performance of these 6 children to that of other (monolingual) children with ASD (N = 24). These two groups did not differ for expressive grammar (*p* = .1), non-verbal reasoning (*p* = .4), or simple and complex-span composite scores (respectively *p* = .9 and *p* = .1). As such, performance for monolinguals and bilinguals is reported as a group.

4 For WM: Forward digit recall, nonword repetition, serial order word span, backward digit recall, counting span. For syntax: root questions and clitics, complex sentence repetition (correct syllables and embedding), and sentence comprehension. For attention: selective attention, processing speed and attention shifting.
