# Supplementary material for: A Preliminary Examination of the Impact of Working Memory Training on Syntax and Processing Speed in Children with ASD
Source: J Autism Dev Disord. 2021 Nov 1;52(10):4233–51. doi: 10.1007/s10803-021-05295-z (PMC8559141; doi:10.1007/s10803-021-05295-z)
Supplement: Supplementary file 1 — Supplementary file1 (DOCX 220 KB) [file 10803_2021_5295_MOESM1_ESM.docx]

**Appendix A.** Detailed participant information

| **Participant** | **Age** | **Gender** | **Nonverbal reasoning** | **Expressive syntax** | **CARS Scores** | **Simple span** | **Complex span** | **Comorbid diagnosis / Specialized education** |
| --- | --- | --- | --- | --- | --- | --- | --- | --- |
| 1 | 8;3 | M | -0.4 | -8.9 | 35,0 | -3.6 | -3.6 |  |
| 2 | 8;7 | M | 0.8 | -7.7 | 38,0 | -2.1 | -2.6 |  |
| 3 | 9;4 | M | -2.3 | -6.7 | 25,0 | -2.1 | -3.1 |  |
| 4 | 9;1 | F | -2.3 | -6.0 | 32,5 | -1.9 | -2.3 |  |
| 5 | 10;4 | M | -1.4 | -5.4 | 34,0 | -0.5 | -2.7 |  |
| 6 | 11;0 | M | -4.2 | -5.3 | 37,0 | -4.1 | -5.0 |  |
| 7 | 11;6 | M | -2.1 | -5.3 | 37,5 | -1.6 | -4.7 |  |
| 8 | 8;7 | M | 0.3 | -5.2 | 26,0 | -0.2 | -2.7 |  |
| 9 | 10;1 | M | 0.2 | -4.8 | 27,0 | -3.1 | -2.4 | Specialized education |
| 10 | 11;10 | M | 0.2 | -4.8 | 24,0 | -1.7 | -4.7 |  |
| 11 | 8;9 | M | 0.4 | -4.8 | 27,0 | -0.9 | -2.6 | ADHD |
| 12 | 9;3 | M | -1.1 | -4.8 | 31,0 | 0.3 | -2.5 |  |
| 13 | 9;5 | M | -3.3 | -4.1 | 34,0 | -1.4 | -1.9 |  |
| 14 | 8;2 | M | -1.3 | -3.9 | 26,0 | -0.4 | -2.6 |  |
| 15 | 9;10 | M | 0.0 | -3.8 | 30,0 | -1.0 | -2.1 | Specialized education |
| 16 | 11;2 | F | -1.9 | -2.9 | 35,0 | -1.1 | -3.0 |  |
| 17 | 7;1 | M | 1.7 | -2.9 | 36,0 | -2.5 | -3.2 |  |
| 18 | 7;7 | M | -0.1 | -2.9 | 23,0 | -0.5 | -2.8 |  |
| 19 | 7;2 | M | 0.3 | -2.7 | 33,5 | -1.1 | -1.6 |  |
| 20 | 8;0 | M | -0.8 | -2.4 | 29,5 | 0.4 | -2.8 |  |
| 21 | 8;5 | M | 0.3 | -2.4 | 29,5 | -1.2 | -1.0 | ADHD |
| 22 | 8;0 | M | -1.5 | -2.2 | 24,5 | -2.5 | -1.9 |  |
| 23 | 6;9 | M | 1.5 | -1.8 | 20,5 | -2.2 | -1.0 |  |
| 24 | 7;11 | M | -1.5 | -1.4 | 23,0 | -0.7 | -1.7 |  |
| 25 | 10;7 | F | -0.5 | -1.4 | 23,0 | 0.3 | -2.2 |  |
| 26 | 6;0 | M | 0.3 | -1.3 | 27,0 | -0.7 | -1.7 |  |
| 27 | 7;3 | M | 0.3 | -1.3 | 23,5 | 1.1 | -1.5 |  |
| 28 | 5;11 | M | -1.4 | -1.3 | 28,0 | -1.1 | -2.2 |  |
| 29 | 6;8 | M | 0.1 | -1.0 | 21,0 | 0.0 | -1.6 |  |
| 30 | 6;9 | M | 1.3 | -1.0 | 24,0 | 0.5 | -1.6 |  |

Interpretation of CARS Scores: < 30 🡪 mild symptoms; 30-36.5 🡪 moderate symptoms; 37-60 🡪 severe symptoms. ADHD: Attention Deficit Hyperactivity Disorder.

**Appendix B.** Complex sentence repetition

| **Syntactic characteristics** | **Sentences** |
| --- | --- |
| **Simple sentence** | **A1.** Le garçon s'amuse avec des petites voitures en bois.  *The boy plays with small wooden cars.*  **B1.** La fille pose ses deux grandes poupées dans son coffre à jouets.  *The girl puts her two large dolls in her toy box.* |
| **0-level relative**  **1 embedding**  **Subject relative** | **A2.** Regarde ! Un homme qui porte un pull et un pantalon bleu.  *Look! A man who is wearing a sweater and blue pants.*  **B2.** Regarde ! Une femme qui tient une valise et un grand chapeau.  *Look! A woman who is holding a suitcase and a big hat.* |
| **0-level relative**  **1 embedding**  **Object relative** | **A3.** Voilà une petite fille que je connais depuis longtemps.  *This is a little girl that I've known for a long time.*  **B3.** Voilà une copine de classe que j’adore depuis toujours.  *This is a school friend that I have always loved.* |
| **Simple sentence** | **A4.** Le petit garçon va à la piscine avec son frère.  *The little boy is going to the swimming pool with his brother.*  **B4.** Le petit chien mange de la viande dans la salle à manger.  *The little dog eats meat in the dining room.* |
| **0-level relative**  **1 embedding**  **Object relative with S-V inversion** | **5A.** Hou là là ! Une petite fille que mord un chien noir et blanc.  *Wow! A little girl that a black and white dog bites.*  **5B.** Hou là là ! Une petite souris que poursuit le chat noir.  *Wow! A little mouse that the black cat is chasing.* |
| **Pseudo-relative**  **1 embedding**  **Subject relative** | **6A.** C'est un garçon qui mange une glace au chocolat au lait.  *It's a boy who is eating a milk chocolate ice cream.*  **6B.** C’est un vieux Monsieur qui fait ses courses au supermarché.  *It's an old man who is shopping at the supermarket.* |
| **Simple sentence** | **7A.** Les enfants regardent la télé dans la salle à manger.  *The children are watching TV in the dining room.*  **7B.** La maman passe l’aspirateur dans la chambre à coucher.  *The mom is vacuuming the bedroom.* |
| **Pseudo-relative**  **1 embedding**  **Object relative** | **8A.** Il y’a une fille qu'elle préfère dans son cours du mercredi.  *There's a girl that she prefers in her Wednesday class.*  **8B.** Il y’a un beau garçon qu’elle aime beaucoup dans son école.  *There is a handsome boy that she likes very much in her school.* |
| **Pseudo-relative**  **1 embedding**  **Object relative with S-V inversion** | **9A.** C'est un chat que caressent tous les enfants après l'école.  *It’s a cat that all of the children pet after school.*  **9B.** C’est le chien que nourrissent les voisins une fois par semaine.  *This is the dog that the neighbors feed once a week.* |
| **Simple sentence** | **10A.** Elle dessine un bonhomme avec des crayons de couleur.  *She draws a man with colored pencils.*  **10B.** Il joue au foot avec un maillot de l’équipe de France.  *He plays soccer with a shirt of the French national team.* |
| **Genuine relative**  **1 embedding**  **Subject relative** | **11A.** La maîtresse voit le garçon qui lit un livre sur Noël.  *The teacher sees the boy who is reading a book about Christmas.*  **11B.** La maîtresse cherche le garçon qui fait ses devoirs de maths.  *The teacher looks for the boy who is doing his math homework.* |
| **Genuine relative**  **1 embedding**  **Object relative** | **12A.** La dame regarde le garçon qu'elle a invité chez elle.  *The lady looks at the boy that she has invited to her home.*  **12B.** La fille aide le garçon qu’elle a fait tomber dans la boue.  *The girl helps the boy that she made fall in the mud.* |
| **Simple sentence** | **13A.** Tous les enfants du village vont à l'école en vélo.  *All the children of the village go to school by bike.*  **13B.** Tous les enfants de l’école vont à la piscine demain.  *All the children in the school are going to the pool tomorrow.* |
| **Genuine relative**  **1 embedding**  **Object relative with S-V inversion** | **14A.** Le papa cherche la grande fille que préfèrent tous les garçons.  *The dad is looking for the tall girl that all the boys prefer.*  **14B.** La maman punit le garçon que détestent toutes les filles.  *The mom punishes the boy that all the girls hate.* |
| **Genuine relative**  **2 embeddings**  **Subject relative** | **15A.** Il pense que son fils aime la maîtresse qui donne des bonnes notes.  *He thinks that his son likes the teacher who gives good marks.*  **15B.** Je pense que le chat aime le garçon qui donne de la viande.  *I think that the cat likes the boy who gives meat.* |
| **Simple sentence** | **16A.** Le monsieur joue au loto avec ses petits-enfants.  *The man plays the lotto with his grandchildren.*  **16B.** La grand-mère joue avec ses petits-enfants aux lego.  *The grandmother plays Legos with her grandchildren.* |
| **Genuine relative**  **2 embeddings**  **Object relative** | **17A.** Je crois que la fille préfère le chien qu'elle a colorié.  *I think that the girl prefers the dog that she colored.*  **17B.** Je crois que la fille déteste les légumes qu’elle a mangés.  *I think that the girl hates the vegetables that she ate.* |
| **Genuine relative**  **2 embeddings**  **Object relative with S-V inversion** | **18A.** Elle dit que Pierre regarde la fille que punit la maîtresse.  *She says that Peter is looking at the girl that the teacher is punishing*.  **18B.** Elle dit que Thomas aime la fille que préfère la maîtresse.  *She says that Thomas loves the girl that the teacher prefers.* |
| **Simple sentence** | **19A.** Le monsieur emmène ses enfants en vacances à la mer.  *The man takes his children on vacation to the sea.*  **19B.** Le grand-père promène ses deux chiens au bord de la rivière.  *The grandfather walks his two dogs by the riverside.* |
| **Genuine relative**  **3 embeddings**  **Subject relative** | **20A.** Il pense qu'elle dit que le garçon déteste la fille qui pleure.  *He thinks that she says that the boy hates the girl who is crying.*  **20B.** Vous pensez qu’elle dit que le fils préfère la dame qui parle.  *You think that she says that the son prefers the lady who is speaking.* |
| **Genuine relative**  **3 embeddings**  **Object relative** | **21A.** Vous dites qu'elle pense que le cheval lèche le chien qu'il préfère ?  *You say that she thinks that the horse licks the dog that it prefers?*  **21B.** Elle dit qu’il pense que le chien lèche le mouton qu’il préfère.  *She says that he thinks that the dog licks the sheep that it prefers.* |
| **Simple sentence** | **22A.** La fille mange des gâteaux au chocolat pour le goûter.  *The girl eats chocolate cakes for the snack.*  **22B.** Le monsieur fume un gros cigare marron sur le balcon.  *The man smokes a big brown cigar on the balcony*. |
| **Genuine relative**  **3 embeddings**  **Object relative with S-V inversion** | **23A.** Je crois qu'il dit que l'ours mord le chien que promène la fille.  *I think that he says that the bear bites the dog that the girl is walking.*  **23B.** Je crois qu’il dit que la vache lèche le chat que porte la fille.  *I think that he says that the cow licks the cat that the girl is carrying.* |

**Appendix C.** Comprehension of complement sentences


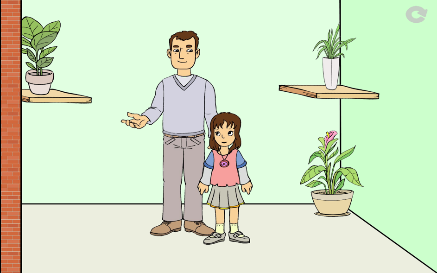

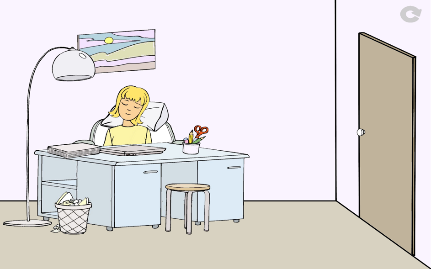

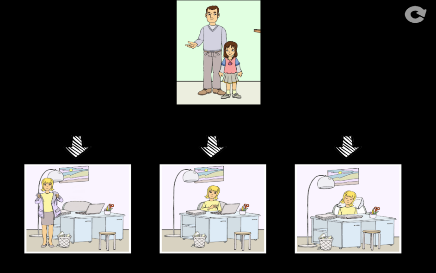


**Appendix D.** Details of working memory training activities

*Activity 1: Serial order memory*

Activity 1, which was inspired by Majerus and colleagues (2006), required participants to retain a series of orally presented verbal items in successive order, with the aim of training serial order short-term memory capacity. After hearing a list of familiar monosyllabic words, participants were asked to place images corresponding to the words in the order in which the words had been presented. The number of heard items increased as the participant’s performance improved, thus adapting the complexity of the task to the child’s ability. Specifically, after two successful attempts at the same span level there was an increase of one item and after two unsuccessful attempts at the same span level there was a decrease of one item.

*Activity 2: WM updating*

Activity 2 is an adapted version of the classic n-back task that we created to train WM updating. The participants were presented with a sequence of visual stimuli and told to tap on the screen of the iPad when the current stimulus matched the one from n steps earlier in the sequence: 1-back, 2-back or 3-back based on the level of difficulty. The task was adaptive as load number adapted automatically to the participant’s ability based on his or her performance on the previous trials: a score of greater than 71% at the end of a session resulted in the increase of the n-back level by one for the subsequent session whereas a score below or equal to 50% resulted in the decrease of the n-back level decreased by one for the next session.

*Activity 3: Serial order and complex WM*

Activity 3, which aimed to train complex span, required participants to store the order of familiar auditory stimuli (e.g. a ringing telephone, a crying baby or a barking dog) while simultaneously performing a visual comparison of quantity task. Participants first listened to a certain number of stimuli while completing a matching number of quantity comparison tasks and were then asked to place images corresponding to the sounds in their order of presentation. The number of presented stimuli increased or decreased throughout the task, on the same basis as in Activity 1.

*Activities 4 and 5: Simple and complex span*

Activities 4 and 5 were designed to train simple and complex WM span. Inspired by the classic digit span task, these activities required participants to retain the order of a series of color names (e.g. blue, red, green). Participants were told to indicate the colors they had heard in order of presentation in Activity 4 and in reverse order in Activity 5. As with Activities 1 and 3, the level of difficulty was tailored to the participant’s ability, increasing or decreasing according to performance on the preceding trials.

**Appendix E:** Individual measures of gains

|  | **Working memory** | | | | | **Syntax** | | | | | **Attention** | | |
| --- | --- | --- | --- | --- | --- | --- | --- | --- | --- | --- | --- | --- | --- |
|  |  |  |  |  |  |  |  | **Repetition** | |  |  |  |  |
| **Participant** | **Forward digit recall** | **Nonword repetition** | **Serial order word span** | **Backward digit recall** | **Counting span** | **Root questions** | **Clitics** | **Syllables** | **Embedding** | **Comprehension** | **Selective attention** | **Processing speed** | **Attention shifting** |
| 1 | 0 | -5 | 4 | 1 | 4 | 4 | -1 | 13 | 0 | 4 | -6.80 | -4.55 | -3.55 |
| 2 | 2 | -3 | 21 | 3 | 9 | -1 | 4 | -39 | -4 | -1 | 0.16 | 0 | -1 |
| 3 | 0 | -4 | 14 | 1 | -7 | 3 | 0 | 9 | -3 | 3 | -1.57 | -1.89 | -17 |
| 4 | 3 | 9 | 13 | 2 | 2 | 2 | 1 | 24 | 2 | 2 | -2.28 | -14 | -45 |
| 5 | 0 | -2 | 19 | 0 | 9 | -3 | -2 | 42 | 1 | -3 | -2.44 | -9 | -11 |
| 6 | 0 | 10 | 12 | 4 | 0 | 3 | 0 | 46 | -1 | 3 | -13.95 | -3.1 | -5 |
| 7 | 1 | -1 | 25 | 4 | 5 | -2 | 0 | -7 | 0 | -2 | 0.77 | -7.75 | -10.89 |
| 8 | 0 | 0 | 7 | 1 | 3 | 1 | -1 | 64 | 4 | 1 | -1.01 | -1 | -25 |
| 9 | 1 | 5 | 12 | 2 | 3 | 2 | 2 | 9 | 3 | 2 | -4.16 | -1 | -3 |
| 10 | 0 | -5 | 16 | 0 | 9 | 5 | 0 | 43 | 0 | 5 | -6.30 | -2.35 | -3.95 |
| 11 | 1 | 10 | -2 | 3 | 21 | 0 | -2 | 0 | 0 | 0 | 1.10 | -6 | -5 |
| 12 | 5 | 6 | 7 | 1 | 8 | 0 | 0 | 3 | 1 | 0 | -2.58 | -3 | -6 |
| 13 | 0 | 18 | 12 | 0 | 0 | -1 | 0 | 9 | 4 | -1 | -0.61 | -5 | -9 |
| 14 | 2 | -4 | 33 | 1 | 2 | 3 | 1 | 0 | 0 | 3 | -1.18 | -4 | -13 |
| 15 | 2 | -4 | 10 | 0 | 10 | -1 | 6 | 11 | 1 | -1 | 6.15 | -14 | -6 |
| 16 | 0 | 27 | 22 | 2 | 1 | -1 | -2 | 11 | -2 | -1 | -1.91 | -2 | -12 |
| 17 | 1 | -4 | 3 | 3 | 0 | 2 | 0 | -4 | -1 | 2 | 0.60 | 1 | 12 |
| 18 | 1 | -3 | 6 | 0 | -1 | 2 | 0 | 75 | 1 | 2 | -12.50 | -2 | -24.1 |
| 19 | 0 | 1 | 7 | 1 | 8 | 4 | 3 | 13 | 2 | 4 | -8.20 | -8 | -7 |
| 20 | -1 | -2 | 5 | 1 | -10 | -4 | 0 | 12 | 1 | -4 | 3.75 | -3 | -2 |
| 21 | 4 | 4 | 18 | 0 | 3 | 1 | 0 | 1 | 4 | 1 | -0.70 | 0 | -2 |
| 22 | 0 | 12 | 25 | 1 | -2 | 1 | -2 | -1 | 0 | 1 | -0.39 | -2 | 0 |
| 23 | 1 | 3 | 7 | 0 | 5 | 1 | 1 | 58 | 2 | 1 | -0.85 | 0 | -1 |
| 24 | 4 | 24 | -1 | 1 | 4 | 0 | 0 | 66 | 5 | 0 | -1.29 | -6 | 1 |
| 25 | -1 | 4 | -4 | -3 | 20 | 2 | 2 | 12 | 1 | 2 | 2.57 | -2.22 | -7.84 |
| 26 | 1 | 5 | 5 | 4 | -2 | 2 | 0 | 20 | 3 | 2 | -5.55 | 5 | -36 |
| 27 | -1 | 16 | -4 | 1 | 6 | 1 | 2 | 11 | -2 | 1 | -0.58 | 8 | 0 |
| 28 | 1 | 39 | 7 | 0 | 0 | -3 | 2 | -1 | -1 | -3 | 1.02 | -33.73 | -19 |
| 29 | 0 | -11 | 11 | 7 | 0 | 2 | -2 | 10 | -1 | 2 | 3.17 | 1 | 2 |
| 30 | 4 | 14 | 22 | 0 | 9 | 8 | 0 | 60 | 4 | 8 | -2.98 | -2 | -4 |
| Mean  (SD) | 1.0  (1.6) | 5.3  (11.1) | 11.1 (9.1) | 1.4  (1.8) | 4.0  (6.5) | 1.1  (2.5) | 0.4  (1.8) | 19.0  (26.2) | 0.8  (2.2) | 1.1  (2.5) | -2.0  (4.3) | -4.1  (7.2) | -8.8  (11.6) |
